# Low Daytime Light Intensity Disrupts Male Copulatory Behavior, and Upregulates Medial Preoptic Area Steroid Hormone and Dopamine Receptor Expression, in a Diurnal Rodent Model of Seasonal Affective Disorder

**DOI:** 10.3389/fnbeh.2019.00072

**Published:** 2019-04-12

**Authors:** Joseph S. Lonstein, Katrina Linning-Duffy, Lily Yan

**Affiliations:** Neuroscience Program & Department of Psychology, Michigan State University, East Lansing, MI, United States

**Keywords:** hormones, light, nucleus accumbens, medial preoptic area, seasonal affective disorder, sexual behavior

## Abstract

Seasonal affective disorder (SAD) involves a number of psychological and behavioral impairments that emerge during the low daytime light intensity associated with winter, but which remit during the high daytime light intensity associated with summer. One symptom frequently reported by SAD patients is reduced sexual interest and activity, but the endocrine and neural bases of this particular impairment during low daylight intensity is unknown. Using a diurnal laboratory rodent, the Nile grass rat (*Arvicanthis niloticus*), we determined how chronic housing under a 12:12 h day/night cycle involving dim low-intensity daylight (50 lux) or bright high-intensity daylight (1,000 lux) affects males’ copulatory behavior, reproductive organ weight, and circulating testosterone. We also examined the expression of mRNAs for the aromatase enzyme, estrogen receptor 1 (ESR1), and androgen receptor (AR) in the medial preoptic area (mPOA; brain site involved in the sensory and hormonal control of copulation), and mRNAs for the dopamine (DA) D1 and D2 receptors in both the mPOA and nucleus accumbens (NAC; brain site involved in stimulus salience and motivation to respond to reward). Compared to male grass rats housed in high-intensity daylight, males in low-intensity daylight displayed fewer mounts and intromissions when interacting with females, but the groups did not differ in their testes or seminal vesicle weights, or in their circulating levels of testosterone. Males in low-intensity daylight unexpectedly had higher ESR1, AR and D1 receptor mRNA in the mPOA, but did not differ from high-intensity daylight males in D1 or D2 mRNA expression in the NAC. Reminiscent of humans with SAD, dim winter-like daylight intensity impairs aspects of sexual behavior in a male diurnal rodent. This effect is not due to reduced circulating testosterone and is associated with upregulation of mPOA steroid and DA receptors that may help maintain some sexual motivation and behavior under winter-like lighting conditions.

## Introduction

Seasonal affective disorder (SAD) is a recurrent major depressive disorder with a seasonal pattern that in most cases worsens in fall and winter, but remits in spring and summer (Rosenthal et al., 1984; American Psychiatric Association, 2013). Up to 5%–10% of people living in latitudes far from the equator are thought to be above the clinical threshold to be diagnosed with SAD, and subsyndromal symptoms are experienced by a much larger percentage of the population (e.g., Potkin et al., 1986; Rosen et al., 1990; Magnusson and Partonen, 2005; Grimaldi et al., 2009; Wirz-Justice et al., 2019). The symptoms of SAD are numerous and include not only depressed mood but also anxiety, irritability, reduced physical activity, hyperphagia, sleep disruption, and low libido (Rosenthal et al., 1984; Jacobsen et al., 1987).

Given the clinical definition of SAD, it is not surprising that most research on seasonal changes in human affect and behavior has focused on the etiology and treatment of depressed mood. There has been considerably less attention paid to the other seasonal changes, and almost none to the biological basis of winter-time decreases in libido, sexual activity, and sexual satisfaction (Kasper et al., 1989; Roenneberg and Aschoff, 1990; Bronson, 1995; Avasthi et al., 2001; Demir et al., 2016; Arendt and Middleton, 2018). Many studies have reported seasonal variation in circulating gonadal steroids in humans (Smals et al., 1976; Ronkainen et al., 1985; Kauppila et al., 1987a,b; Kivelä et al., 1988; Dabbs, 1990; Meriggiola et al., 1996; Valero-Politi and Fuentes-Arderiu, 1998; Garde et al., 2000; Wisniewski and Nelson, 2000; van Anders et al., 2006; Stanton et al., 2011; Demir et al., 2016), but there is no evidence that SAD patients have atypical gonadal hormone levels at any time of year (although they do for some pituitary and adrenal hormones—Jacobsen et al., 1987; Avery et al., 1997; Martiny et al., 2004; Thorn et al., 2011). In addition, the seasonal changes in testosterone particularly seen in men most often involve a peak in fall/winter (Smals et al., 1976; Dabbs, 1990; Valero-Politi and Fuentes-Arderiu, 1998; Wisniewski and Nelson, 2000; van Anders et al., 2006; Stanton et al., 2011), which does not temporally correspond with what would be expected for a wintertime decline in sexual interest and activity. Thus, the low winter-time libido and sexual function in SAD patients and other individuals in the general population is unlikely to be due to a drop in circulating gonadal hormone levels. Interestingly, there is some evidence that the winter-time reduction in libido also does not depend on the presence of a mood disorder (Bossini et al., 2009).

The underlying mechanisms may instead be due to seasonal modifications in the central nervous system sites underlying sexual behaviors. The neural network involved in mammalian sexual behaviors includes the medial preoptic area (mPOA) lying just rostral to the hypothalamus. In many animals, the mPOA is critical for the sensory and gonadal steroid regulation of partner choice, sexual motivation, and/or the expression of copulatory behaviors (for reviews see Hull and Dominguez, 2015; Micevych and Meisel, 2017; Pfaff and Baum, 2018). Relevant to SAD, the hormonal sensitivity of the mPOA is affected by changes in season or photoperiod. Winter-like short day length reduces mPOA aromatase activity (the enzyme that converts testosterone into estradiol) in seasonally breeding male Golden hamsters (Callard et al., 1986), causes a drop in androgen receptor (AR) binding in their mPOA (Bittman and Krey, 1988), decreases AR immunoreactivity in the mPOA of male Siberian hamsters (Tetel et al., 2004), and lowers both estrogen receptor (ESR) and progestin receptor immunoreactivity in the mPOA of female Syrian hamsters (Mangels et al., 1998). Similar effects of the season can be found for the steroid hormone sensitivity of the mPOA in sheep (Skinner and Herbison, 1997) and European starlings (Riters et al., 2000).

Not only is the mPOA’s response to steroid hormones critical for its role in the display of sexual behaviors, but activity of the neurotransmitter, dopamine (DA), in the mPOA is also essential. DA is released in the mPOA of male rats and Japanese quail when they are exposed to female sensory cues, and this DA response appears to determine their subsequent behavioral interactions with the female (Hull et al., 1995; Kleitz-Nelson et al., 2010). Disrupting DA receptor signaling in the mPOA by infusing the D1/D2 receptor antagonist, cis-flupenthixol, impairs both sexual motivation and performance in male rats (Pehek et al., 1988). Furthermore, selective antagonism or agonism of D1 and D2 receptors in the mPOA reveals that low D1 signaling and high D2 signaling is especially disruptive for males’ latency to begin copulating, but hastens ejaculation (Hull et al., 1989). D1 and D2 signaling in the mPOA also modulates sexual behaviors in female rats, with high D1 or D2 activity promoting sexual solicitation depending on the subjects’ ovarian hormone milieu (Graham and Pfaus, 2010, 2012). Lastly, the consolidation of sexual experience in male rats requires mPOA D1 receptor activity during interactions with the female (McHenry et al., 2012), while others have shown that previous sexual experience not only increases the number of D2 receptor-immunoreactive cells in the male rat mPOA but also that Fos expression in these D2R-immunoreactive cells is positively correlated with a number of facets of their copulatory behaviors (Nutsch et al., 2016).

The mPOA is not the only site in the brain where changes in DA signaling may be associated with seasonal changes in libido and sexual activity. Similar to other depressive disorders, SAD involves decreased interest or pleasure in most activities (i.e., anhedonia; American Psychiatric Association, 2013), which is associated with the mesolimbic DA system dysfunction (Nestler and Carlezon, 2006). Mesolimbic DA signaling is essential for high sexual motivation and behaviors in laboratory rodents (Yoest et al., 2014; Hull and Dominguez, 2015), and natural or experimental changes in ambient light do alter DA synthesis, metabolism, and receptor content in many areas of the laboratory rodent and human brain (e.g., Neumeister et al., 2001; Eisenberg et al., 2010; Tsai et al., 2011; Cawley et al., 2013; Deats et al., 2015; Goda et al., 2015; Itzhacki et al., 2018).

It is reasonable to hypothesize that decreased libido in male SAD patients results from altered gonadal steroid and DA sensitivity of the mPOA and nucleus accumbens (NAC). In the present experiment, we tested this hypothesis in a male diurnal rodent—the Nile grass rat (*Arvicanthis noliticus*). We have found that, similar to SAD patients, winter-like lighting regimen (involving either short daylength or reduced daytime light intensity) increases depression- and anxiety-like behaviors and produces cognitive impairments in Nile grass rats (Leach et al., 2013a,b; Deats et al., 2014; Ikeno et al., 2016; Soler et al., 2018). Those behavioral responses are accompanied by changes in central DA, serotonin, orexin, neurotrophin, and stress-mediating systems (Leach et al., 2013a,b; Deats et al., 2014; Ikeno et al., 2016; Soler et al., 2018). Humans are diurnal and neurobehaviorally stimulated by light, so studying diurnal grass rats offers advantages for understanding how light affects the human brain and behavior over studying most other laboratory rodents (which are nocturnal; Yan et al., 2019). Also similar to humans, but not some laboratory rodents like hamsters that are seasonal breeders, grass rats will copulate all year around (McElhinny et al., 1997; Blanchong et al., 1999) so are an excellent model for studying seasonal influences on diurnal male copulatory behavior, testosterone levels, and brains.

Instead of studying the effects of day length or daylight duration, we here studied the effects of winter-like daylight intensity. This is because it is the seasonal differences in daylight intensity that are most salient to modern humans. Most humans around the world now use artificial lights, so the duration of light across seasons is much less variable than the intensity of the light we receive across seasons (Hébert et al., 1998). We predicted that housing in winter-like, low-intensity daylight would: (1) impair males’ copulatory behaviors when paired with a conspecific female; (2) decrease aromatase, ESR1, AR, D1 and D2 mRNA expression in the mPOA; and (3) reduce D1 and D2 mRNA expression in the NAC.

## Materials and Methods

### Subjects

Subjects were from a stock of Nile grass rats (*Arvicanthus nilotocus*) originally captured in sub-Saharan Africa by Dr. Laura Smale in 1993 and maintained for almost two decades at Michigan State University using outbred breeding (McElhinny et al., 1997). Animals were housed in transparent polypropylene cages (43 × 23 × 20 cm) containing wood chip bedding and an 8 × 6 cm PVC pipe for shelter/enrichment. They were maintained under 12 h light-12 h dark conditions (lights on at 06:00 h), typical of their equatorial habitat. After reaching adulthood, males were singly housed. Pre-experimental colony room light was supplied by cool white fluorescent lights mounted on the ceiling, with light intensity at the center of the room at ~300 lux. Animals had food (Prolab 2000 #5P06, PMI Nutrition) and water *ad libitum*. This study was carried out in accordance with the recommendations of the National Institutes of Health Guide for the Care and Use of Laboratory Animals (NIH Publication No. 80-23). The protocol was approved by the Institutional Animal Care and Use Committee of Michigan State University.

### Lighting Conditions

At the onset of the experiment, males were moved out of the colony and singly housed in smaller environmental chambers. In these chambers, they were subjected to a 12 h bright daylight (1,000 lux)-12 h dark (1 lux) condition (bright Light/Dark; brLD) or a 12 h dim daylight (50 lux)-12 h dark (1 lux) condition (dim Light/Dark; dimLD) for 5 weeks (for sexual behavior tests) or for 4 weeks (for testosterone, reproductive organ, and brain measures). Different groups of animals were used for the behavioral and biological measures in order to avoid any effects of group-level differences in sexual experience complicating the biological data interpretation. Our prior work has shown that male grass rats housed in brLD and dimLD for 4–5 weeks differ in their anxiety- and depression-related behaviors, stress responsiveness, spatial memory, and numerous neural characteristics (Leach et al., 2013a,b; Deats et al., 2014; Ikeno et al., 2016). Light was provided by cool white fluorescent bulbs (Jesco Lighting, SP4-26SW/30-W), with the same full spectrum maintained in both the brLD and dimLD conditions.

### Copulatory Behavior

Before being placed into brLD or dimLD conditions, males were screened to ensure they copulated during a 30-min interaction with unfamiliar female grass rats from our colony that were primed with subcutaneous injections of 10 or 20 μg estradiol benzoate once a day for two consecutive days, followed 24 h later by a subcutaneous injection of 250 or 500 μg progesterone (Sigma, USA). Females were used for behavior testing starting 3 h later. Males were removed from their home cages and placed in larger glass aquaria (61 × 32 × 29 cm) that contained wood chips for bedding and a hormone-primed female under ~300 lux (i.e., colony room) illumination. Males that successfully copulated were then randomly assigned to be housed in either the brLD (*n* = 11) or the dimLD (*n* = 14) condition for 5 weeks. After 5 weeks, they were then tested again in the glass aquaria for copulatory behaviors with unfamiliar ovarian hormone-primed female grass rats. If males did not make contact with a stimulus female within 2 min after the beginning testing, or if a stimulus female did not show lordosis in response to a male’s mounts, the stimulus female was replaced with another hormone-primed female grass rat and the test started over. Male-female interactions were recorded for 15 min and males’ latencies to begin sniffing the females, latencies to first mount, their frequencies of mounts, and their frequencies of intromissions were later scored. “Hit rate” as a measure of copulatory efficiency was determined by the number of intromissions divided by the number of mounts plus intromissions × 100. Ejaculations were not reliably observed in most males within the 15-min observations.

### Reproductive Organ Weights and Plasma Testosterone Levels

A set of experimentally naïve animals from the colony were housed in either brLD (*n* = 7) or dimLD (*n* = 8) for 4 weeks and sacrificed between 09:00 and 10:00 h with an overdose of sodium pentobarbital. Animals were weighed, their testes and seminal vesicles collected and stripped of fat, and the fresh tissues weighed to the nearest mg. Trunk blood was obtained from another set of experimentally naïve male grass rats housed in each condition for 4 weeks and sacrificed during the day at Zeitgeber T2 (nine dimLD, nine brLD) or in the evening at T14 (eight dimLD, eight brLD). Blood was centrifuged at 15,000 rpm for 15 min, and the plasma stored at −80°C until being assayed for testosterone using a commercially available EIA kit per the manufacturer’s protocol (Enzo Life Sciences, Farmingdale, NY, USA).

### Brain Processing and qtPCR

Brains from experimentally naïve male grass rats (brLD *n* = 11 for mPOA, *n* = 10 for NAC; dimLD *n* = 8 for mPOA; *n* = 12 for NAC) were collected, quickly frozen on dry ice, and stored at −80°C until later analysis of five mRNAs relevant to sexual and other motivated behaviors. Brains were cut into 200-μm sections and bilateral 1-mm diameter micropunches (Harris Micropunch, Electron Microscopy Sciences, Pennsylvania, PA, USA) were made to obtain the mPOA and NAC. Tissue was processed and qtPCR run as described previously (Grieb et al., 2018). Briefly, tissue punches were homogenized by pulsed sonication in RLT Plus buffer (Qiagen, Germantown, MD, USA) containing β-mercaptoethanol. mRNAs were extracted using an RNeasy Plus Mini Kit (Qiagen, Germantown, MD, USA) and quantified with a GeneQuant100 machine (Harvard Bioscience, Holliston, MA, USA). RNA purity was determined by the ratio of absorbances at 260:280 nm wavelengths, and ratios of ~2.0 was considered pure. Equal concentrations of mRNAs were converted to cDNA using a high-capacity reverse transcription kit (Applied Biosystems, Foster City, CA, USA). Real-time PCR was conducted with 2.5 ng/μl of converted cDNA (based on starting concentration of RNA converted to cDNA) and samples were run in triplicate. Runs included cDNA, primers, and SYBR Green PCR Master Mix (Applied Biosystems, Foster City, CA, USA) in a 25-μL reaction involving an ABI PRISM 7000 Sequence Detection System (Applied Biosystems, Foster City, CA, USA). A no-template control was run alongside the samples to ensure that no primer-dimer amplification had occurred. In addition, mRNA samples not run through the reverse transcription kit were also run at the same time to ensure no gDNA contamination. Amplification efficiencies were calculated for each primer set, and each was within the accepted range (1.90–2.10) to use the ΔΔCT method to calculate fold change between groups (Schmittgen and Livak, 2008).

Preoptic area mRNAs analyzed were for the androgen receptor (forward—ACTACTTCTCTGCAGTGCCT; reverse—CCAGGAAATAGAACTGGGGAAC), ESR1 (forward—CCAGCTCCACTTCAGCACAT; reverse—GAGCCTGGGAGTTCTCAGAT), and aromatase (forward—CTACTGTCTGGGAATCGGGC; reverse—GTTGCAGGCACTTCCAATCC). mPOA and NAC mRNAs analyzed were for the DA D1 receptor (forward—GTGGGCGAATTCTTCCCTGA; reverse—GGGCAGAGTCTGTAGCATCC) and D2 receptor (forward—GGACATGAAACTCTGCACCG; reverse—ATCCATTCTCCGCCTGTTCAC). All were compared to the “housekeeping” gene HPRT1 (forward—CTCATGGACTGATTATGGACAGGAC; reverse-GCAGGTCAGCAAAGAACTTATAGCC). The primers (Integrated DNA Technologies, Coralville, IA, USA) were designed based on the corresponding gene sequences in laboratory mice and rats, and the identities of all the PCR products we obtained in grass rats were confirmed by sequencing at the MSU Genomic Core.

### Data Analyses

Data sets were confirmed to be normally distributed and have homogeneous variance among groups. Data were subjected to Grubb’s single-outlier tests and any outliers were removed. The data were then analyzed using two-tailed independent Student *t*-tests comparing groups of brLD males to dimLD males. Statistical significance was indicated by *p*s < 0.05.

## Results

### Copulatory Behaviors

**Figure 1:**
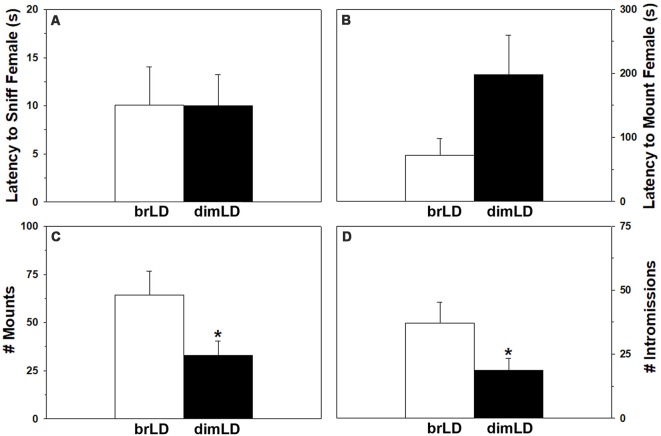
Behavioral response to dim daylight. Male grass rats were housed in bright daylight intensity (brLD) or dim daylight intensity (dimLD) and their **(A)** latency to sniff an estrus female, **(B)** latency to mount an estrus female, **(C)** frequency of mounts, and **(D)** frequency of intromissions is shown (Means ± SEMs). *p < 0.05.

All brLD and dimLD male grass rats included in the study copulated with ovarian hormone-primed stimulus females during the 15-min observations. The groups did not significantly differ in their latency to begin sniffing the females (*t*_(23)_ = 0.18, *p* = 0.986), but there was a trend for dimLD males to take longer to mount (*t*_(23)_ = 1.704, *p* = 0.10). DimLD males also mounted the females less frequently (*t*_(23)_ = 2.28, *p* < 0.033) and intromitted less often (*t*_(23)_ = 2.10 *p* = 0.047) compared to brLD males (Figure 1). Males’ “hit rate” was similar between the brLD and dimLD groups (*t*_(23)_ = 0.30, *p* = 0.766).

### Reproductive Organs and Plasma Testosterone

**Figure 2:**
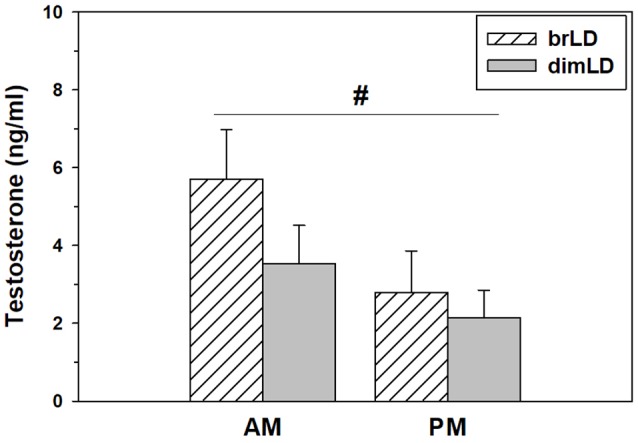
Hormonal response to dim daylight. Circulating levels (Mean ± SEM) of testosterone in male grass rats housed in bright daylight intensity condition (brLD) or dim daylight intensity (dimLD) condition, and sacrificed during the day (hatched) or night (gray). ^#^Significant main effect of time of day, *p* < 0.05.

Daytime light intensity did not affect males’ testes weights (1.40 ± 0.03 vs. 1.45 ± 0.44 g/g bodyweight × 100; *t*_(13)_ = 0.96, *p* = 0.35) or seminal vesicle weights (0.91 ± 0.12 vs. 0.89 ± 0.07 g/g bodyweight × 100; *t*_(13)_ = 0.18, *p* = 0.85). It also did not affect their levels of circulating testosterone (*F*_(1,34)_ = 1.83, *p* = 0.18), although there was a significant effect of when blood was taken, such that male grass rats sacrificed during the day had higher circulating testosterone compared to males sacrificed at night (*F*_(1,34)_ = 4.23, *p* = 0.049; Figure 2). There was no significant interaction between light intensity group and time of day on testosterone levels (*F*_(1,34)_ = 0.528, *p* = 0.47).

### Medial Preoptic Area and Nucleus Accumbens mRNAs

**Figure 3:**
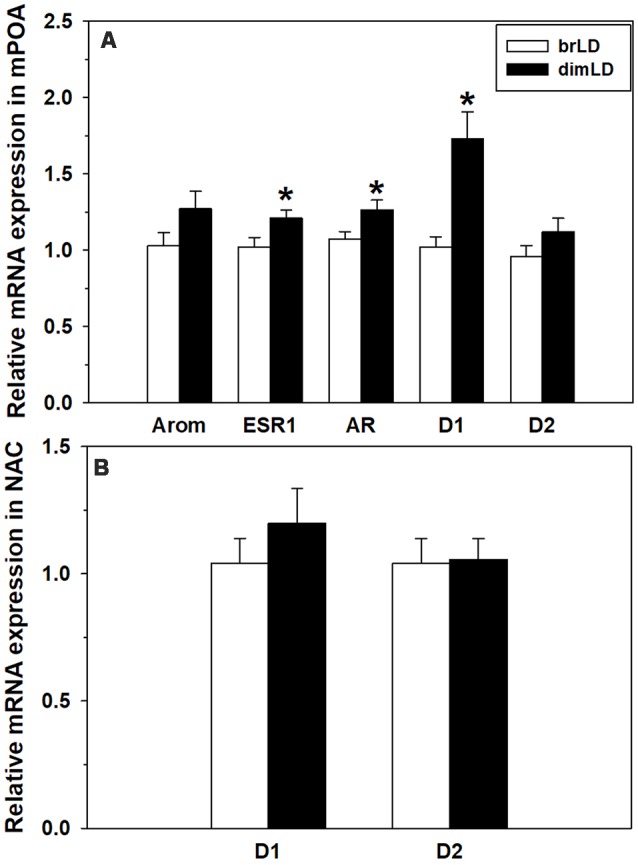
Neural response to dim daylight. **(A)** Relative levels (Mean ± SEM) of mRNAs for aromatase (Arom), estrogen receptor 1 (ESR1), androgen receptor (AR), D1 and D2 in the medial preoptic area (mPOA) of male grass rats housed in bright daylight intensity condition (brLD) or dim daylight intensity (dimLD) condition. **(B)** Relative levels (Mean ± SEM) of mRNAs for D1 and D2 in the nucleus accumbens (NAC) of male grass rats housed in brLD or dimLD conditions. *p < 0.05.

Male grass rats housed in brLD and dimLD conditions did not differ in their mPOA aromatase mRNA expression (*t*_(17)_ = 1.635, *p* = 0.120), but dimLD males had significantly higher ESR1 (*t*_(17)_ = 2.175, *p* = 0.044) and AR (*t*_(16)_ = 2.31, *p* = 0.035) mRNA expression compared to brLD males (Figure 3A). dimLD males also had significantly higher D1 (*t*_(17)_ = 4.21, *p* = 0.001), but not D2 (*t*_(16)_ = 1.52, *p* = 0.149), receptor mRNA expression in the mPOA. In the NAC, dimLD and brLD males did not significantly differ in their DA D1 (*t*_(20)_ = 0.874, *p* = 0.393) or D2 (*t*_(19)_ = 0.129, *p* = 0.878) receptor mRNA expression (Figure 3B).

## Discussion

Winter-time decrease in libido, sexual activity, and sexual satisfaction are commonly associated with SAD, and also experienced by many people whose seasonal symptoms would not reach the clinical threshold for this disorder (Schlager et al., 1993; Harmatz et al., 2000; Avasthi et al., 2001; Demir et al., 2016; Arendt and Middleton, 2018). It has been suggested that the seasonal decrease in sexual function (and thus mating) may have evolutionary origins, such that births would be less likely to occur during the increasingly resource-poor autumn, and instead be biased toward early spring (Eagles, 2004; Davis and Levitan, 2005). In support, SAD is more common in people of reproductive age than those younger or older (Magnusson et al., 2000). Despite a possible evolutionary benefit, for most modern humans around the globe which use artificial light and live in resource-rich environments, seasonal changes in sexual function can be quite distressing and very little is known about the neurobiological underpinnings.

### Influence of Daytime Light Intensity on Male Copulatory Behavior and Relevance to SAD

Using diurnal male grass rats as a model, we hypothesized that housing in low-intensity light during the daytime would recapitulate the effects of winter on male sexual activities. Our results generally supported this hypothesis. When compared to males housed in the bright, high-intensity daylight condition (brLD), males in the dim, low-intensity daylight condition (dimLD) had a trend toward longer latencies to begin mounting estrus females and showed about half as many mounts and intromissions. This suggests that, in male grass rats, being housed in winter-like daylight intensity may have only minor negative effects on sexual approach/motivation (reflected by the latencies to sniff and mount the females), but more considerable disruptive effects on copulatory performance (mounts and intromission). This is consistent with the winter-time reduction in men’s sexual activity and satisfaction reported in a number of studies (Schlager et al., 1993; Harmatz et al., 2000; Avasthi et al., 2001; Demir et al., 2016; Arendt and Middleton, 2018).

At first, however, our results appear somewhat inconsistent with the reduced libido (i.e., sexual interest and motivation) often thought to be associated with SAD (Rosenthal et al., 1984). While no animal model is likely to reflect all symptoms of a given disorder, we also believe there is a lack of clarity in the “low libido” often cited as a symptom of SAD. First, the diagnostic criteria for SAD in the DSM-5 do not specifically involve anything related to sexual motivation or performance. Although some instruments frequently used to examine the depressive symptoms associated with SAD do ask a question about sexual functioning, they either offer libido as the only example of a concern related to sexual activity for patients to endorse (HRDS/HAM-D), only ask specifically about libido (BDI), or only ask about sexual satisfaction (Zung’s SDS). Other common instruments do not ask about sexuality at all (CES-D, HADS-Dl QUIDS-SR16, MADRS, SPAQ). Thus, it is unclear if seasonal changes in the frequency or satisfaction of sexual activity are or are not usually captured by the high endorsement of “low libido” in SAD patients. Greater discrimination among the motivational, behavioral, and emotional aspects of human sexual functioning in SAD would be useful for understanding the validity of our and other laboratory rodent models of this affective disorder.

### Influence of Daytime Light Intensity on Gonadal Function

Short winter-like day length increases the duration of melatonin released from the pineal gland each day. Particularly in animals that are seasonal breeders, this elevated melatonin signal inhibits pituitary gonadotropin synthesis, causes gonadal regression, and eventually the cessation of mating (Carter and Goldman, 1983; Arendt, 1986; Kriegsfeld et al., 2015; Weems et al., 2015; Simonneaux, 2019). Of note, short day lengths do not reduce circulating testosterone in some laboratory rodents that are not highly seasonal breeders including rats (Prendergast and Kay, 2008), CD1 mice (Nelson, 1990), and California mice (*P. californicus*; Trainor et al., 2008; also see Trainor et al., 2006). Short day lengths also do not affect testosterone levels or testes mass in male grass rats housed within the laboratory (Nunes et al., 2002). As far as we are aware, there have been no previous studies on the effects of winter-like daylight intensity on gonadal function in any diurnal or nocturnal rodent. We found that male grass rats housed in brLD or dimLD conditions had similar circulating testosterone levels that were within the range previously reported for laboratory-housed and wild male grass rats (Sicard et al., 1994; Nunes et al., 2002). Testes and seminal vesicle weights were also similar between our two groups. We did not have an *a priori* expectation for these measures because it is unclear if grass rats are somewhat seasonal breeders or simply opportunistic breeders that mostly rely on recent environmental conditions to determine their reproduction (Neal, 1981; Delany and Monro, 1986; Sicard et al., 1994). It should be kept in mind for interpreting our results that the behavioral data and the blood (and brains) were obtained from different groups of dimLD and brLD male grass rats, which was done to avoid confounding effects of group differences in copulatory behaviors on circulating testosterone. Also, the sets of dimLD and brLD males used for behavior and blood analyses differed by 1 week in how long they were in their lighting conditions, although there are no obvious reasons why a difference of 4 vs. 5 weeks would have mattered for males’ circulating gonadal hormones.

In any case, our results suggest that the impairments in copulatory behavior in dimLD-housed males are independent of their gonadal function. Other studies have found that changes in laboratory rodent behavior due to day length are also not positively related to gonadal hormones. For example, aggression in female Siberian hamsters housed in short day lengths is independent of their circulating ovarian hormones (Scotti et al., 2007), and aggression in male Siberian hamsters is inversely related to their circulating testosterone (Jasnow et al., 2000). In male California mice, housing in short days does not affect circulating testosterone (Nelson et al., 1995), but still increases aggression (Trainor et al., 2008). In laboratory rats, short-day lengths increase depression- and anxiety-like behaviors (Prendergast and Kay, 2008; although see Dulcis et al., 2013) without affecting circulating testosterone or testes size (Wallen et al., 1987; Prendergast and Kay, 2008). It has also long been known that in some subpopulations of male laboratory rodents there is no positive relationship between their circulating testosterone and copulatory behaviors (Davidson, 1966; Damassa et al., 1977). Additionally, as mentioned above there is no evidence that gonadal function is abnormal in men who experience winter-time reductions in libido, sexual activity, or sexual satisfaction; our results from male grass rats suggest that these reductions are likely unrelated to circulating testicular hormones.

While we found no effect of daytime light intensity on gonadal function, we did find significantly higher circulating testosterone in male grass rats sacrificed in the morning compared to those sacrificed at night. A circadian rhythm in circulating gonadal hormones is commonly found in mammals (Kriegsfeld et al., 2002), and the circadian rhythm suggested by our results is similar to the morning peak in testosterone found in diurnal humans (e.g., Diver et al., 2003; Brambilla et al., 2009). It is also consistent with the early-morning peak and evening nadir in hypothalamic gonadotropin releasing hormone (GnRH) cell activity, and circulating luteinizing hormone (LH), in female grass rats from our colony (McElhinny et al., 1999). In contrast to diurnal grass rats, the nocturnal laboratory rat and mouse show their peak circulating testosterone levels in the early evening (Kriegsfeld et al., 2015).

### Influence of Daytime Light Intensity on mPOA Steroid Hormone and DA Receptor mRNAs

The mPOA is a critical node in the neural network underlying the hormonal and sensory control of male sexual behavior (Hull and Dominguez, 2015). There is a large population of cells in the mPOA where endogenous testosterone can be aromatized into estradiol, which then acts on cytosolic ESRs as well as other substrates to regulate male sexual motivation and performance (Balthazart et al., 2004). Testosterone also acts directly on ARs in the mPOA to promote male sexual activity (McGinnis et al., 2002; Harding and McGinnis, 2004). We found that mPOA levels of aromatase mRNA did not differ between males housed in dimLD and brLD conditions, suggesting a similar capacity for local estradiol synthesis. The groups differed in ESR1 and AR mRNA expression, though, with both transcripts higher in dimLD males than brLD males. This was unexpected because the impaired copulation in the dimLD-housed males would have intuitively been consistent with lower ESR1 and AR expression in the mPOA. Thus rendering it less sensitive to steroid hormone influences on sexual behavior.

Winter-like short day length downregulates ARs in the mPOA of seasonally breeding male Golden and Siberian hamsters (Bittman and Krey, 1988; Tetel et al., 2004), as well as downregulates ESR1 in the mPOA of female Syrian hamsters (Mangels et al., 1998), concomitant with cessation of their copulatory behavior. In non-seasonal breeders, however, the consequences of short day length on central AR and ER expression are more complex, being both species- and site-specific (Trainor et al., 2007, 2008). Similar to our findings related to reduced daytime light intensity, short day length increases ESR1 mRNA in the mPOA of male Oldfield mice (*P. polionotus*). However, housing in short days does not affect ESR1 mRNA expression in the mPOA of male Deer mice (*P. maniculatus*; Trainor et al., 2007) or estrogen receptor immunoreactivity in the mPOA of Siberian hamsters (Kramer et al., 2008). Short day length also increases ESR1 mRNA and/or estrogen receptor protein in the posterior bed nucleus of the stria terminalis (BNST) of male Oldfield mice, Deer mice, and Siberian hamsters, but not in male California mice (*P. californicus*; Trainor et al., 2007, 2008; Kramer et al., 2008). Because these non-seasonally breeding animals show no changes in circulating testosterone or sexual behavior in response to short day length, the seasonal changes in their behavior (e.g., increased aggression and anxiety) are independent of circulating steroids. In our male grass rats, reduced copulatory behavior in response to low-intensity daylight is also independent of circulating testosterone and more likely associated in some manner with the upregulated AR and ESR1 expression in their mPOA. Given the direction of the results, we propose that the upregulated expression may be part of a compensatory mechanism that maintains sexual behavior, albeit at lower levels, in the dimLD males. An alternative hypothesis could be that upregulation of these receptors in the mPOA actively suppresses male copulatory behavior in dimLD males. While we can find no evidence from the literature that upregulating AR or ESR1 in the mPOA would impair copulation or any other sociosexual behavior in male rats, overexpressing estrogen receptors in other sites such as the medial amygdala or BNST does reduce prosocial behaviors in male prairie voles (Cushing et al., 2008; Lei et al., 2010).

We also found upregulated D1 mRNA in the mPOA of dimLD-housed male grass rats, which was unexpected based on what is known about DA receptors in this brain site and male copulation. High mPOA D1 activity relative to D2 activity is associated with faster sexual motivation and more mounts and intromissions before ejaculation in male laboratory rats (Hull et al., 1989; Moses et al., 1995), not the somewhat longer latencies to mount females and lower mount and intromissions frequencies in our dimLD grass rats. Similar to the upregulated AR and ESR1 expression in the mPOA, perhaps upregulated D1 receptor expression in the mPOA of dimLD male grass rats helps maintain some level of sexual activity in these animals.

### Influence of Daytime Light Intensity on NAC DA Receptor mRNAs

DA receptor signaling in the NAC is essential for incentive salience, motivation to approach, and/or continued responding for a rewarding stimulus (Salamone et al., 2005; Castro and Berridge, 2014). It would make sense if that extends to natural rewards such as sexual activity, but the literature so far has shown that neither D1 or D2 antagonism nor D2 agonism in the NAC affects copulatory behavior in male laboratory rats tested under high-motivation conditions (Hull et al., 1986; Moses et al., 1995; Guadarrama-Bazante and Rodríguez-Manzo, 2019). Nonetheless, D2 agonism in the NAC (but not mPOA) can reinstate mounting by sexually sated male rats that have low motivation to copulate (Guadarrama-Bazante and Rodríguez-Manzo, 2019).

We found no significant difference between our two groups of male grass rats in levels of D1 or D2 receptor mRNAs in the NAC. This does not mean that other aspects of their mesolimbic systems do not differ in ways that affect their sexual behavior, and this would be worthy of future investigation. A number of studies report relationships among light, DA, and mood. Pharmacologically induced catecholamine depletion during the summer causes relapse in SAD patients (Lam et al., 2001) and significantly lowers mood in healthy women living for just 2 days under a normal circadian cycle but with very dim daytime light intensity (10 lux; Cawley et al., 2013). A study of people naturally experiencing winter-time short day length along with low daytime light intensity found that the ventral tegmental area and other DA-rich midbrain cell groups have much less tyrosine hydroxylase immunoreactivity compared to midbrains obtained from people during the summer (Aumann et al., 2016). On the other hand, human cerebrospinal fluid levels of DA and its metabolites are higher (Hartikainen et al., 1991), striatal presynaptic DA synthesis and storage are elevated (Eisenberg et al., 2010), and DA transporter binding in the striatum is lower (Neumeister et al., 2001) in winter. Even the small differences in potential sunshine exposure in a subtropical location (i.e., Taiwan) is associated with lower D2/D3 receptor availability in the human striatum (Tsai et al., 2011). These results collectively suggest a winter-time downregulation of midbrain DA cells, but upregulation of forebrain terminal and synaptic mechanisms that may permit some degree of compensation in humans. The effects of winter-like conditions on central DA systems in diurnal rodents is less clear. Male Nile grass rats in either winter-like photoperiod or low daytime light intensity have fewer hypothalamic cells containing tyrosine hydroxylase (Deats et al., 2015) and winter-like reduction in both daylight length and intensity downregulate DA turnover in the NAC of Sudanian grass rats (*Arvicanthis* ansorgei), which could be restored to control levels by daily bright light “therapy” (Itzhacki et al., 2018). In diurnal chipmunks, short days decreased DA content in the striatum but increased DA levels in the hypothalamus and amygdala (Goda et al., 2015).

## Conclusions and Future Directions

Seasonal changes in human sexual motivation and function are quite common but are rarely studied. As such, the biological bases are poorly understood. The present findings from diurnal male grass rats, along with other research on diurnal and non-seasonally breeding rodents, indicates that the locus of such effects of winter-time light conditions is not at the level of the gonads but in brain sites such as the mPOA that are vital for sexual activity. It will be valuable in future studies to determine if such effects of dim daylight on sexual behavior in grass rats are reversible by daily “light therapy.” It will also be valuable to determine the effects of winter-like dim daylight on copulation, ovarian hormone levels, and relevant mRNAs in the mPOA of female grass rats. Women suffer from SAD 2–4 times more often men (Kasper et al., 1989; Lee and Chan, 1998), and consistently show some inhibition of ovarian function during winter (Ronkainen et al., 1985; Kauppila et al., 1987a,b; Kivelä et al., 1988). Perhaps female grass rats housed in dim daylight would show less copulatory behaviors compared to females in bright daylight, but the effects are due to both a drop in ovarian function and reduced steroid and DA sensitivity of their mPOA and other hypothalamic sites involved in female copulation (e.g., ventromedial nucleus).

## Ethics Statement

This study was carried out in accordance with the recommendations of the National Institutes of Health Guide for the Care and Use of Laboratory Animals (NIH Publication No. 80-23). The protocol was approved by the Institutional Animal Care and Use Committee of Michigan State University.

## Author Contributions

JL and LY conceived and designed the experiments. KL-D conducted the experiments. JL, LY, and KL-D conducted data analyses and wrote the manuscript.

## Conflict of Interest Statement

The authors declare that the research was conducted in the absence of any commercial or financial relationships that could be construed as a potential conflict of interest.

## References

[B1] American Psychiatric Association (2013). Diagnostic and Statistical Manual of Mental Disorders. 5th Edn. Arlington, VA: American Psychiatric Publishing.

[B3] Arendt J. (1986). Role of the pineal gland and melatonin in seasonal reproductive function in mammals. Oxf. Rev. Reprod. Biol. 8, 266–320. 3540805

[B2] Arendt J. Middleton B. (2018). Human seasonal and circadian studies in Antarctica (Halley, 75°S). Gen. Comp. Endocrinol. 258, 250–258. 10.1016/j.ygcen.2017.05.010 28526480

[B4] Aumann T. D. Raabus M. Tomas D. Prijanto A. Churilov L. Spitzer N. C.. (2016). Differences in number of midbrain dopamine neurons associated with summer and winter photoperiods in humans. PLoS One 11:e0158847. 10.1371/journal.pone.0158847 27428306 PMC4948786

[B5] Avasthi A. Sharma A. Gupta N. Kulhara P. Varma V. K. Malhotra S.. (2001). Seasonality and affective disorders: a report from North India. J. Affect. Disord. 64, 145–154. 10.1016/s0165-0327(00)00239-1 11313081

[B6] Avery D. H. Dahl K. Savage M. V. Brengelmann G. L. Larsen L. H. Kenny M. A.. (1997). Circadian temperature and cortisol rhythms during a constant routine are phase-delayed in hypersomnic winter depression. Biol. Psychiatry 41, 1109–1123. 10.1016/s0006-3223(96)00210-7 9146822

[B7] Balthazart J. Baillien M. Cornil C. A. Ball G. F. (2004). Preoptic aromatase modulates male sexual behavior: slow and fast mechanisms of action. Physiol. Behav. 83, 247–270. 10.1016/j.physbeh.2004.08.025 15488543

[B8] Bittman E. L. Krey L. C. (1988). Influences of photoperiod on nuclear androgen receptor occupancy in neuroendocrine tissues of the golden hamster. Neuroendocrinology 47, 61–67. 10.1159/000124892 3277081

[B9] Blanchong J. A. McElhinny T. L. Mahoney M. M. Smale L. (1999). Nocturnal and diurnal rhythms in the unstriped Nile rat, *Arvicanthis niloticus*. J. Biol. Rhythms 14, 364–377. 10.1177/074873099129000777 10511004

[B10] Bossini L. Fagiolini A. Valdagno M. Roggi M. Tallis V. Trovarelli S.. (2009). Light therapy as a treatment for sexual dysfunctions. Psychother. Psychosom. 78, 127–128. 10.1159/000203119 19223689

[B11] Brambilla D. J. Matsumoto A. M. Araujo A. B. McKinlay J. B. (2009). The effect of diurnal variation on clinical measurement of serum testosterone and other sex hormone levels in men. J. Clin. Endocrinol. Metab. 94, 907–913. 10.1210/jc.2008-1902 19088162 PMC2681273

[B12] Bronson F. H. (1995). Seasonal variation in human reproduction: environmental factors. Q. Rev. Biol. 70, 141–164. 10.1086/418980 7610233

[B13] Callard G. V. Mak P. Solomon D. J. (1986). Effects of short days on aromatization and accumulation of nuclear estrogen receptors in the hamster brain. Biol. Reprod. 35, 282–291. 10.1095/biolreprod35.2.282 3768455

[B14] Carter D. S. Goldman B. D. (1983). Progonadal role of the pineal in the Djungarian hamster (*Phodopus sungorus* sungorus): mediation by melatonin. Endocrinology 113, 1268–1273. 10.1210/endo-113-4-1268 6413193

[B15] Castro D. C. Berridge K. C. (2014). Advances in the neurobiological bases for food ‘liking’ versus ‘wanting’. Physiol. Behav. 136, 22–30. 10.1016/j.physbeh.2014.05.022 24874776 PMC4246030

[B16] Cawley E. I. Park S. aan het Rot M. Sancton K. Benkelfat C. Young S. N.. (2013). Dopamine and light: dissecting effects on mood and motivational states in women with subsyndromal seasonal affective disorder. J. Psychiatry Neurosci. 38, 388–397. 10.1503/jpn.120181 23735584 PMC3819153

[B17] Cushing B. S. Perry A. Musatov S. Ogawa S. Papademetriou E. (2008). Estrogen receptors in the medial amygdala inhibit the expression of male prosocial behavior. J. Neurosci. 28, 10399–10403. 10.1523/jneurosci.1928-08.2008 18842899 PMC2586115

[B18] Dabbs J. M. Jr. (1990). Age and seasonal variation in serum testosterone concentration among men. Chronobiol. Int. 7, 245–249. 10.3109/07420529009056982 2268886

[B19] Damassa D. A. Smith E. R. Tennent B. Davidson J. M. (1977). The relationship between circulating testosterone levels and male sexual behavior in rats. Horm. Behav. 8, 275–286. 10.1016/0018-506x(77)90002-2 881168

[B20] Davidson J. M. (1966). Characteristics of sex behaviour in male rats following castration. Anim. Behav. 14, 266–272. 10.1016/s0003-3472(66)80082-9 5956590

[B21] Davis C. Levitan R. D. (2005). Seasonality and seasonal affective disorder (SAD): an evolutionary viewpoint tied to energy conservation and reproductive cycles. J. Affect. Disord. 87, 3–10. 10.1016/j.jad.2005.03.006 15927269

[B22] Deats S. P. Adidharma W. Lonstein J. S. Yan L. (2014). Attenuated orexinergic signaling underlies depression-like responses induced by daytime light deficiency. Neuroscience 272, 252–260. 10.1016/j.neuroscience.2014.04.069 24813431 PMC4090246

[B23] Deats S. P. Adidharma W. Yan L. (2015). Hypothalamic dopaminergic neurons in an animal model of seasonal affective disorder. Neurosci. Lett. 602, 17–21. 10.1016/j.neulet.2015.06.038 26116821 PMC4532597

[B24] Delany M. J. Monro R. H. (1986). Population dynamics of *Arvicanthis niloticus* (Rodentia: Muridae) in Kenya. J. Zool. 209, 85–103. 10.1111/j.1469-7998.1986.tb03567.x

[B25] Demir A. Uslu M. Arslan O. E. (2016). The effect of seasonal variation on sexual behaviors in males and its correlation with hormone levels: a prospective clinical trial. Cent. European J. Urol. 69, 285–289. 10.5173/ceju.2016.838 27729996 PMC5057046

[B27] Diver M. J. Imtiaz K. E. Ahmad A. M. Vora J. P. Fraser W. D. (2003). Diurnal rhythms of serum total, free and bioavailable testosterone and of SHBG in middle-aged men compared with those in young men. Clin. Endocrinol. 58, 710–717. 10.1046/j.1365-2265.2003.01772.x 12780747

[B28] Dulcis D. Jamshidi P. Leutgeb S. Spitzer N. C. (2013). Neurotransmitter switching in the adult brain regulates behavior. Science 340, 449–453. 10.1126/science.1234152 23620046

[B29] Eagles J. M. (2004). Seasonal affective disorder: a vestigial evolutionary advantage? Med. Hypotheses 63, 767–772. 10.1016/j.mehy.2004.07.002 15488644

[B30] Eisenberg D. P. Kohn P. D. Baller E. B. Bronstein J. A. Masdeu J. C. Berman K. F. (2010). Seasonal effects on human striatal presynaptic dopamine synthesis. J. Neurosci. 30, 14691–14694. 10.1523/jneurosci.1953-10.2010 21048126 PMC3010858

[B31] Garde A. H. Hansen A. M. Skovgaard L. T. Christensen J. M. (2000). Seasonal and biological variation of blood concentrations of total cholesterol, dehydroepiandrosterone sulfate, hemoglobin A(1c), IgA, prolactin and free testosterone in healthy women. Clin. Chem. 46, 551–559. 10759480

[B32] Goda R. Otsuka T. Iwamoto A. Kawai M. Shibata S. Furuse M.. (2015). Serotonin levels in the dorsal raphe nuclei of both chipmunks and mice are enhanced by long photoperiod, but brain dopamine level response to photoperiod is species-specific. Neurosci. Lett. 593, 95–100. 10.1016/j.neulet.2015.03.035 25797183

[B33] Graham M. D. Pfaus J. G. (2010). Differential regulation of female sexual behaviour by dopamine agonists in the medial preoptic area. Pharmacol. Biochem. Behav. 97, 284–292. 10.1016/j.pbb.2010.08.012 20807549

[B34] Graham M. D. Pfaus J. G. (2012). Differential effects of dopamine antagonists infused to the medial preoptic area on the sexual behavior of female rats primed with estrogen and progesterone. Pharmacol. Biochem. Behav. 102, 532–539. 10.1016/j.pbb.2012.06.020 22750065

[B35] Grieb Z. A. Holschbach M. A. Lonstein J. S. (2018). Interaction between postpartum stage and litter age on maternal caregiving and medial preoptic area orexin. Physiol. Behav. 194, 430–436. 10.1016/j.physbeh.2018.06.025 29928888 PMC6089372

[B36] Grimaldi S. Partonen T. Haukka J. Aromaa A. Lönnqvist J. (2009). Seasonal vegetative and affective symptoms in the Finnish general population: testing the dual vulnerability and latitude effect hypotheses. Nord. J. Psychiatry 63, 397–404. 10.1080/08039480902878729 19363741

[B37] Guadarrama-Bazante I. L. Rodríguez-Manzo G. (2019). Nucleus accumbens dopamine increases sexual motivation in sexually satiated male rats. Psychopharmacology [Epub ahead of print]. 10.1007/s00213-018-5142-y 30536080

[B38] Harding S. M. McGinnis M. Y. (2004). Androgen receptor blockade in the MPOA or VMN: effects on male sociosexual behaviors. Physiol. Behav. 81, 671–680. 10.1016/j.physbeh.2004.03.008 15178162

[B39] Harmatz M. G. Well A. D. Overtree C. E. Kawamura K. Y. Rosal M. Ockene I. S. (2000). Seasonal variation of depression and other moods: a longitudinal approach. J. Biol. Rhythms 15, 344–350. 10.1177/074873000129001350 10942266

[B40] Hartikainen P. Soininen H. Reinikainen K. J. Sirviö J. Soikkeli R. Riekkinen P. J. (1991). Neurotransmitter markers in the cerebrospinal fluid of normal subjects. Effects of aging and other confounding factors. J. Neural Transm. Gen. Sect. 84, 103–117. 10.1007/bf01249114 1675857

[B41] Hébert M. Dumont M. Paquet J. (1998). Seasonal and diurnal patterns of human illumination under natural conditions. Chronobiol. Int. 15, 59–70. 10.3109/07420529808998670 9493715

[B42] Hull E. M. Bitran D. Pehek E. A. Warner R. K. Band L. C. Holmes G. M. (1986). Dopaminergic control of male sex behavior in rats: effects of an intracerebrally-infused agonist. Brain Res. 370, 73–81. 10.1016/0006-8993(86)91106-6 3011196

[B43] Hull E. M. Dominguez J. M. (2015). “Male sexual behavior,” in Knobil and Neill’s Physiology of Reproduction, eds Plant T. M. Zeleznik A. J. (New York, NY: Elsevier), 2211–2285.

[B44] Hull E. M. Du J. Lorrain D. S. Matuszewich L. (1995). Extracellular dopamine in the medial preoptic area: implications for sexual motivation and hormonal control of copulation. J. Neurosci. 15, 7465–7471. 10.1523/JNEUROSCI.15-11-07465.1995 7472498 PMC6578034

[B45] Hull E. M. Warner R. K. Bazzett T. J. Eaton R. C. Thompson J. T. Scaletta L. L. (1989). D2/D1 ratio in the medial preoptic area affects copulation of male rats. J. Pharmacol. Exp. Ther. 251, 422–427. 2572689

[B46] Ikeno T. Deats S. P. Soler J. Lonstein J. S. Yan L. (2016). Decreased daytime illumination leads to anxiety-like behaviors and HPA axis dysregulation in the diurnal grass rat (*Arvicanthis niloticus*). Behav. Brain Res. 300, 77–84. 10.1016/j.bbr.2015.12.004 26684510 PMC4724441

[B47] Itzhacki J. Clesse D. Goumon Y. Van Someren E. J. Mendoza J. (2018). Light rescues circadian behavior and brain dopamine abnormalities in diurnal rodents exposed to a winter-like photoperiod. Brain Struct. Funct. 223, 2641–2652. 10.1007/s00429-018-1655-8 29560509

[B48] Jacobsen F. M. Wehr T. A. Sack D. A. James S. P. Rosenthal N. E. (1987). Seasonal affective disorder: a review of the syndrome and its public health implications. Am. J. Public Health 77, 57–60. 10.2105/ajph.77.1.57 3789239 PMC1646810

[B49] Jasnow A. M. Huhman K. L. Bartness T. J. Demas G. E. (2000). Short-day increases in aggression are inversely related to circulating testosterone concentrations in male Siberian hamsters (*Phodopus sungorus*). Horm. Behav. 38, 102–110. 10.1006/hbeh.2000.1604 10964524

[B50] Kasper S. Wehr T. A. Bartko J. J. Gaist P. A. Rosenthal N. E. (1989). Epidemiological findings of seasonal changes in mood and behavior. Arch. Gen. Psychiatry 46, 823–833. 10.1001/archpsyc.1989.01810090065010 2789026

[B51] Kauppila A. Kivelä A. Pakarinen A. Vakkuri O. (1987a). Inverse seasonal relationship between melatonin and ovarian activity in humans in a region with a strong seasonal contrast in luminosity. J. Clin. Endocrinol. Metab. 65, 823–828. 10.1210/jcem-65-5-823 3667880

[B52] Kauppila A. Pakarinen A. Kirkinen P. Mäkilä U. (1987b). The effect of season on the circulating concentrations of anterior pituitary, ovarian and adrenal cortex hormones and hormone binding proteins in the subarctic area; evidence of increased activity of the pituitary- ovarian axis in spring. Gynecol. Endocrinol. 1, 137–150. 10.3109/09513598709030678 3140579

[B53] Kivelä A. Kauppila A. Ylöstalo P. Vakkuri O. Leppäluoto J. (1988). Seasonal, menstrual and circadian secretions of melatonin, gonadotropins and prolactin in women. Acta Physiol. Scand. 132, 321–327. 10.1111/j.1748-1716.1988.tb08335.x 3147572

[B54] Kleitz-Nelson H. K. Dominguez J. M. Cornil C. A. Ball G. F. (2010). Is sexual motivational state linked to dopamine release in the medial preoptic area? Behav. Neurosci. 124, 300–304. 10.1037/a0018767 20364890 PMC2852173

[B55] Kramer K. M. Simmons J. L. Freeman D. A. (2008). Photoperiod alters central distribution of estrogen receptor α in brain regions that regulate aggression. Horm. Behav. 53, 358–365. 10.1016/j.yhbeh.2007.11.002 18078937

[B56] Kriegsfeld L. J. LeSauter J. Hamada T. Pitts S. M. Silver R. (2002). “Circadian rhythms in the endocrine system,” in Hormones, Brain and Behavior, eds Pfaff D. W. Arnold A. P. Etgen A. M. Rubin R. T. (New York, NY: Academic Press), 33–91.

[B57] Kriegsfeld L. J. Ubuka T. Bentley G. E. Tsutsui K. (2015). Seasonal control of gonadotropin- inhibitory hormone (GnIH) in birds and mammals. Front. Neuroendocrinol. 37, 65–75. 10.1016/j.yfrne.2014.12.001 25511257 PMC4405439

[B58] Lam R. W. Tam E. M. Grewal A. Yatham L. N. (2001). Effects of α-methyl-para-tyrosine- induced catecholamine depletion in patients with seasonal affective disorder in summer remission. Neuropsychopharmacology 25, S97–S101. 10.1016/s0893-133x(01)00337-2 11682283

[B59] Leach G. Adidharma W. Yan L. (2013a). Depression-like responses induced by daytime light deficiency in the diurnal grass rat (*Arvicanthis niloticus*). PLoS One 8:e57115. 10.1371/journal.pone.0057115 23437327 PMC3577787

[B60] Leach G. Ramanathan C. Langel J. Yan L. (2013b). Responses of brain and behavior to changing day length in the diurnal grass rat (*Arvicanthis niloticus*). Neuroscience 234, 31–39. 10.1016/j.neuroscience.2013.01.002 23313227 PMC3594136

[B61] Lee T. M. Chan C. C. (1998). Vulnerability by sex to seasonal affective disorder. Percept. Motor Skills 87, 1020–1022. 10.2466/pms.1998.87.3.1120 9885084

[B62] Lei K. Cushing B. S. Musatov S. Ogawa S. Kramer K. M. (2010). Estrogen receptor-α in the bed nucleus of the stria terminalis regulates social affiliation in male prairie voles (*Microtus ochrogaster*). PLoS One 5:e8931. 10.1371/journal.pone.0008931 20111713 PMC2811737

[B63] Magnusson A. Axelsson J. Karlsson M. M. Oskarsson H. (2000). Lack of seasonal mood change in the Icelandic population: results of a cross-sectional study. Am. J. Psychiatry 157, 234–238. 10.1176/appi.ajp.157.2.234 10671392

[B64] Magnusson A. Partonen T. (2005). The diagnosis, symptomatology, and epidemiology of seasonal affective disorder. CNS Spectr. 10, 625–634. 10.1017/s1092852900019593 16041294

[B65] Mangels R. A. Powers J. B. Blaustein J. D. (1998). Effect of photoperiod on neural estrogen and progestin receptor immunoreactivity in female Syrian hamsters. Brain Res. 796, 63–74. 10.1016/s0006-8993(98)00318-7 9689455

[B66] Martiny K. Simonsen C. Lunde M. Clemmensen L. Bech P. (2004). Decreasing TSH levels in patients with Seasonal Affective Disorder (SAD) responding to 1 week of bright light therapy. J. Affect. Disord. 79, 253–257. 10.1016/s0165-0327(02)00361-0 15023503

[B67] McElhinny T. L. Sisk C. L. Holekamp K. E. Smale L. (1999). A morning surge in plasma luteinizing hormone coincides with elevated c-Fos expression in gonadotropin-releasing hormone-immunoreactive neurons in the diurnal rodent, *Arvicanthis niloticus*. Biol. Reprod. 61, 1115–1122. 10.1095/biolreprod61.4.1115 10491652

[B68] McElhinny T. L. Smale L. Holekamp K. E. (1997). Patterns of body temperature, activity, and reproductive behavior in a tropical murid rodent, *Arvicanthis niloticus*. Physiol. Behav. 62, 91–96. 10.1016/s0031-9384(97)00146-7 9226347

[B69] McGinnis M. Y. Montana R. C. Lumia A. R. (2002). Effects of hydroxyflutamide in the medial preoptic area or lateral septum on reproductive behaviors in male rats. Brain Res. Bull. 59, 227–234. 10.1016/s0361-9230(02)00869-9 12431753

[B70] McHenry J. A. Bell G. A. Parrish B. P. Hull E. M. (2012). Dopamine D1 receptors and phosphorylation of dopamine- and cyclic AMP-regulated phosphoprotein-32 in the medial preoptic area are involved in experience-induced enhancement of male sexual behavior in rats. Behav. Neurosci. 126, 523–529. 10.1037/a0028707 22708956 PMC3409344

[B71] Meriggiola M. C. Noonan E. A. Paulsen C. A. Bremner W. J. (1996). Annual patterns of luteinizing hormone, follicle stimulating hormone, testosterone and inhibin in normal men. Hum. Reprod. 11, 248–252. 10.1093/humrep/11.2.248 8671203

[B72] Micevych P. E. Meisel R. L. (2017). Integrating neural circuits controlling female sexual behavior. Front. Syst. Neurosci. 11:42. 10.3389/fnsys.2017.00042 28642689 PMC5462959

[B73] Moses J. Loucks J. A. Watson H. L. Matuszewich L. Hull E. M. (1995). Dopaminergic drugs in the medial preoptic area and nucleus accumbens: effects on motor activity, sexual motivation, and sexual performance. Pharmacol. Biochem. Behav. 51, 681–686. 10.1016/0091-3057(94)00437-n 7675843

[B74] Neal B. R. (1981). Reproductive biology of the unstriped grass rat, *Arvicanthis*, in East Africa. Z. Sauget. 46, 174–189.

[B76] Nelson R. J. (1990). Photoperiodic responsiveness in house mice. Physiol. Behav. 48, 403–408. 10.1016/0031-9384(90)90335-2 2267249

[B75] Nelson R. J. Gubernick D. J. Blom J. M. (1995). Influence of photoperiod, green food and water availability on reproduction in male California mice (*Peromyscus californicus*). Physiol. Behav. 57, 1175–1180. 10.1016/0031-9384(94)00380-n 7652040

[B77] Nestler E. J. Carlezon W. A. (2006). The mesolimbic dopamine reward circuit in depression. Biol. Psychiatry 59, 1151–1159. 10.1016/j.biopsych.2005.09.018 16566899

[B78] Neumeister A. Willeit M. Praschak-Rieder N. Asenbaum S. Stastny J. Hilger E.. (2001). Dopamine transporter availability in symptomatic depressed patients with seasonal affective disorder and healthy controls. Psychol. Med. 31, 1467–1473. 10.1017/s003329170105434z 11722161

[B79] Nunes S. McElhinny T. L. Mahoney M. M. Smale L. (2002). Effects of photoperiod on the reproductive condition of Nile grass rats (*Arvicanthis niloticus*) from an equatorial population. Afr. J. Ecol. 40, 295–302. 10.1046/j.1365-2028.2002.00378.x

[B80] Nutsch V. L. Will R. G. Robison C. L. Martz J. R. Tobiansky D. J. Dominguez J. M. (2016). Colocali- zation of mating-induced Fos and D2-like dopamine receptors in the medial preoptic area: influence of sexual experience. Front. Behav. Neurosci. 10:75. 10.3389/fnbeh.2016.00075 27147996 PMC4834303

[B81] Pehek E. A. Warner R. K. Bazzett T. J. Bitran D. Band L. C. Eaton R. C.. (1988). Microinjection of cis-flupenthixol, a dopamine antagonist, into the medial preoptic area impairs sexual behavior of male rats. Brain Res. 443, 70–76. 10.1016/0006-8993(88)91599-5 3359281

[B82] Pfaff D. W. Baum M. J. (2018). Hormone-dependent medial preoptic/lumbar spinal cord/autonomic coordination supporting male sexual behaviors. Mol. Cell. Endocrinol. 467, 21–30. 10.1016/j.mce.2017.10.018 29100889

[B83] Potkin S. G. Zetin M. Stamenkovic V. Kripke D. Bunney W. E. Jr. (1986). Seasonal affective disorder: prevalence varies with latitude and climate. Clin. Neuropharmacol. 9, 181–183. 3567938

[B84] Prendergast B. J. Kay L. M. (2008). Affective and adrenocorticotrophic responses to photoperiod in Wistar rats. J. Neuroendocrinol. 20, 261–267. 10.1111/j.1365-2826.2007.01633.x 18047552

[B85] Riters L. V. Eens M. Pinxten R. Duffy D. L. Balthazart J. Ball G. F. (2000). Seasonal changes in courtship song and the medial preoptic area in male European starlings (*Sturnus vulgaris*). Horm. Behav. 38, 250–261. 10.1006/hbeh.2000.1623 11104643

[B86] Roenneberg T. Aschoff J. (1990). Annual rhythm of human reproduction: I. Biology, sociology, or both? J. Biol. Rhythms 5, 195–216. 10.1177/074873049000500303 2133132

[B87] Ronkainen H. Pakarinen A. Kirkinen P. Kauppila A. (1985). Physical exercise-induced changes and season-associated differences in the pituitary-ovarian function of runners and joggers. J. Clin. Endocrinol. Metab. 60, 416–422. 10.1210/jcem-60-3-416 3919040

[B88] Rosen L. N. Targum S. D. Terman M. Bryant M. J. Hoffman H. Kasper S. F.. (1990). Prevalence of seasonal affective disorder at four latitudes. Psychiatry Res. 31, 131–144. 10.1016/0165-1781(90)90116-m 2326393

[B89] Rosenthal N. E. Sack D. A. Gillin J. C. Lewy A. J. Goodwin F. K. Davenport Y.. (1984). Seasonal affective disorder. A description of the syndrome and preliminary findings with light therapy. Arch. Gen. Psychiatry 41, 72–80. 10.1001/archpsyc.1984.01790120076010 6581756

[B90] Salamone J. D. Correa M. Mingote S. M. Weber S. M. (2005). Beyond the reward hypothesis: alternative functions of nucleus accumbens dopamine. Curr. Opin. Pharmacol. 5, 34–41. 10.1016/j.coph.2004.09.004 15661623

[B91] Schlager D. Schwartz J. E. Bromet E. J. (1993). Seasonal variations of current symptoms in a healthy population. Br. J. Psychiatry 163, 322–326. 10.1192/bjp.163.3.322 8401960

[B92] Schmittgen T. D. Livak K. J. (2008). Analyzing real-time PCR data by the comparative C_T_ method. Nat. Protoc. 3, 1101–1108. 10.1038/nprot.2008.73 18546601

[B93] Scotti M. A. Place N. J. Demas G. E. (2007). Short-day increases in aggression are independent of circulating gonadal steroids in female Siberian hamsters (*Phodopus sungorus*). Horm. Behav. 52, 183–190. 10.1016/j.yhbeh.2007.03.029 17499250

[B94] Sicard B. Maurel D. Fuminier F. Boissin J. (1994). Climate, trophic factors, and breeding patterns of the Nile grass rat (*Arvicanthis niloticus* solatus): a 5-year study in the Sahelian region of Burkina Faso (formerly Upper Volta). Can. J. Zool. 72, 201–214. 10.1139/z94-027

[B95] Simonneaux V. (2019). A kiss to drive rhythms in reproduction. Eur. J. Neurosci. [Epub ahead of print]. 10.1111/ejn.14287 30472752

[B96] Skinner D. C. Herbison A. E. (1997). Effects of photoperiod on estrogen receptor, tyrosine hydroxylase, neuropeptide Y, and β-endorphin immunoreactivity in the ewe hypothalamus. Endocrinology 138, 2585–2595. 10.1210/endo.138.6.5208 9165052

[B97] Smals A. G. Kloppenborg P. W. Benraad T. J. (1976). Circannual cycle in plasma testosterone levels in man. J. Clin. Endocrinol. Metab. 42, 979–982. 10.1210/jcem-42-5-979 1270587

[B98] Soler J. E. Robison A. J. Núñez A. A. Yan L. (2018). Light modulates hippocampal function and spatial learning in a diurnal rodent species: a study using male Nile grass rat (*Arvicanthis niloticus*). Hippocampus 28, 189–200. 10.1002/hipo.22822 29251803 PMC5820160

[B99] Stanton S. J. Mullette-Gillman O. A. Huettel S. A. (2011). Seasonal variation of salivary testosterone in men, normally cycling women, and women using hormonal contraceptives. Physiol. Behav. 104, 804–808. 10.1016/j.physbeh.2011.07.009 21802437 PMC3494403

[B100] Tetel M. J. Ungar T. C. Hassan B. Bittman E. L. (2004). Photoperiodic regulation of androgen receptor and steroid receptor coactivator-1 in Siberian hamster brain. Mol. Brain Res. 131, 79–87. 10.1016/j.molbrainres.2004.08.009 15530655 PMC2692347

[B101] Thorn L. Evans P. Cannon A. Hucklebridge F. Clow A. (2011). Seasonal differences in the diurnal pattern of cortisol secretion in healthy participants and those with self-assessed seasonal affective disorder. Psychoneuroendocrinology 36, 816–823. 10.1016/j.psyneuen.2010.11.003 21145663

[B103] Trainor B. C. Finy M. S. Nelson R. J. (2008). Rapid effects of estradiol on male aggression depend on photoperiod in reproductively non-responsive mice. Horm. Behav. 53, 192–199. 10.1016/j.yhbeh.2007.09.016 17976598 PMC2190085

[B105] Trainor B. C. Martin L. B. II. Greiwe K. M. Kuhlman J. R. Nelson R. J. (2006). Social and photoperiod effects on reproduction in five species of *Peromyscus*. Gen. Comp. Endocrinol. 148, 252–259. 10.1016/j.ygcen.2006.03.006 16626709 PMC2080678

[B106] Trainor B. C. Rowland M. R. Nelson R. J. (2007). Photoperiod affects estrogen receptor α, estrogen receptor β and aggressive behavior. Eur. J. Neurosci. 26, 207–218. 10.1111/j.1460-9568.2007.05654.x 17614949 PMC2071923

[B107] Tsai H. Y. Chen K. C. Yang Y. K. Chen P. S. Yeh T. L. Chiu N. T.. (2011). Sunshine-exposure variation of human striatal dopamine D_2_/D_3_ receptor availability in healthy volunteers. Prog. Neuropsychopharmacol. Biol. Psychiatry 35, 107–110. 10.1016/j.pnpbp.2010.09.014 20875835

[B108] Valero-Politi J. Fuentes-Arderiu X. (1998). Annual rhythmic variations of follitropin, lutropin, testosterone and sex-hormone-binding globulin in men. Clin. Chim. Acta 271, 57–71. 10.1016/s0009-8981(97)00239-8 9564557

[B109] van Anders S. M. Hampson E. Watson N. V. (2006). Seasonality, waist-to-hip ratio, and salivary testosterone. Psychoneuroendocrinology 31, 895–899. 10.1016/j.psyneuen.2006.03.002 16675146

[B110] Wallen E. P. DeRosch M. A. Thebert A. Losee-Olson S. Turek F. W. (1987). Photoperiodic response in the male laboratory rat. Biol. Reprod. 37, 22–27. 10.1095/biolreprod37.1.22 3651546

[B111] Weems P. W. Goodman R. L. Lehman M. N. (2015). Neural mechanisms controlling seasonal reproduction: principles derived from the sheep model and its comparison with hamsters. Front. Neuroendocrinol. 37, 43–51. 10.1016/j.yfrne.2014.12.002 25582913 PMC4405450

[B112] Wirz-Justice A. Ajdacic V. Rössler W. Steinhausen H. C. Angst J. (2019). Prevalence of seasonal depression in a prospective cohort study. Eur. Arch. Psychiatry Clin. Neurosci. [Epub ahead of print]. 10.1007/s00406-018-0921-3 30022319

[B113] Wisniewski A. B. Nelson R. J. (2000). Seasonal variation in human functional cerebral lateralization and free testosterone concentrations. Brain Cogn. 43, 429–438. 10857741

[B114] Yan L. Lonstein J. S. Nunez A. A. (2019). Light as a modulator of emotion and cognition: lessons learned from studying a diurnal rodent. Horm. Behav. [Epub ahead of print]. 10.1016/j.yhbeh.2018.09.003 30244030 PMC6456444

[B115] Yoest K. E. Cummings J. A. Becker J. B. (2014). Estradiol, dopamine and motivation. Cent. Nerv. Syst. Agents Med. Chem. 14, 83–89. 10.2174/1871524914666141226103135 25540977 PMC4793919

